# Surprising Differences in the Practice of Exclusive Breastfeeding in Non-Roma and Roma Population in Serbia

**DOI:** 10.3389/fpubh.2020.00277

**Published:** 2020-06-30

**Authors:** Zeljka Stamenkovic, Bojana Matejic, Bosiljka Djikanovic, Vesna Bjegovic-Mikanovic

**Affiliations:** Institute of Social Medicine, Medical Faculty University of Belgrade, Belgrade, Serbia

**Keywords:** breastfeeding, Roma, non-Roma, baby friendly, childbirth preparation program

## Abstract

**Background:** Exclusive breastfeeding is essential for early childhood development, although the use of adaptive milk formulas instead of breastfeeding is widespread nowadays. This study aimed to examine the prevalence of exclusively breastfed infants under the age of 6 months in non-Roma and Roma population and factors associated with this practice.

**Materials and Methods:** This study is a secondary analysis of the Serbian Multiple Indicator Cluster Survey investigating non-Roma and Roma infants under the age of 6 months. The study included mothers of 321 non-Roma and 164 Roma infants younger than 6 months. Univariate and multivariate logistic regression served to analyze factors associated with the practice of exclusive breastfeeding in both populations.

**Results:** The prevalence of exclusive breastfeeding was almost the same among mothers in both non-Roma and Roma population (13.3 vs. 13%, *p* = 0.910). Exclusive breastfeeding was significantly more often (*p* < 0.001) among wealthier women, women whose newborns were over 2,500 g on birth, multipara, and women who had not established menstrual cycle among both populations. Living outside the capital significantly diminishes the chance for exclusively breastfed infants in the non-Roma community (Vojvodina: OR 0.16, CI 95% 0.03–0.92; eastern Serbia: OR 0.02, CI 95% 0.01–0.35) as well as living in the rural area (urban: OR 10.35, CI 95% 1.94–55.28). Unexpectedly, in the non-Roma population, not staying in the same room with the newborn in the maternity ward increases the chance for the baby to be exclusively breastfed (OR 7.19, CI 95% 1.80–28.68). The same pattern has been observed in Roma population. Non-Roma mothers multipara are more likely to exclusively breastfeed their children than primipara (OR 7.78, CI 95% 1.09–20.93), while among Roma mothers, the inverse association has been found although not significant (OR 0.42, CI 95% 0.14–1.23). Attending a childbirth preparation program more than 18 times increases the chances of infants being exclusively breastfed (OR 18.65, CI 95% 1.34–53.67). In the Roma population, there was no single woman that attended a childbirth preparation program.

**Conclusion:** The pattern of exclusive breastfeeding significantly differs between non-Roma and Roma populations. Preventive work should have focus on strengthening support to mothers and medical staff in maternity wards.

## Introduction

Breastfeeding and breast milk represent the standard of nutrition for babies that improves the health of mothers and children throughout life (1, 2). While breastfeeding is vital for early childhood development, mothers also enjoy significant health benefits of breastfeeding in terms of establishing an emotional relationship with the child, reducing the risk of postpartum depression, postpartum bleeding and anemia, and long-term effects such as the risk reduction for osteoporosis, malignant breast and ovaries diseases and type 2 diabetes (1–3).

The Global Strategy on Infant and Young Child Feeding clearly defines the optimal nutritional parameters for infants and young children. The strategy recommends breastfeeding to be initiated during the first hour after birth and that the infant should be exclusively breastfed during the first 6 months of life—which means there are no other foods or liquids, including water (1, 2).

Globally, only 40% of children under the age of 6 months are exclusively breastfed (4). Therefore, one of six major global nutritional goals in World Health Organization (WHO) Comprehensive Nutrition, Mothers and Infants Plan is the increase of exclusive breastfeeding practice during the first 6 months to at least 50% (5).

The health system and the analysis of its simulative and dissimulative practices play a significant role in the promotion of breastfeeding. These practices refer to the initiation of breastfeeding in the first hour after delivery or feeding with some non-breast milk in the maternity ward. Whether a child is born in a hospital or outside a hospital, in a rural or urban area, placing newborns on the breast in the first hour after birth gives them the best chance of surviving, establishing long-term breastfeeding, growing and developing to full potential (1, 2, 6). Skin contact immediately after birth increases the likelihood that babies will be breastfed during the first months of life and also prolongs the duration of breastfeeding (6).

WHO and The United Nations Children's Fund (UNICEF) launched the Baby-Friendly Hospital Initiative (BFHI) to improve the breastfeeding practices and support breastfeeding in maternity wards worldwide. Ten Steps to Successful Breastfeeding are the guideline of the initiative, and it emphasizes the importance of early breastfeeding and achieving optimal breastfeeding practices (7, 8). A systematic review of the Baby-friendly Hospital Initiative in 19 countries has shown that adherence to the Ten Steps to Successful Breastfeeding can increase breastfeeding rates (9).

Initiation and length of breastfeeding depend on demographics, biological and socioeconomic factors, the impact of the community, and public health policy (10). Demographic and socioeconomic factors can cause breastfeeding cessation before 6 months of the infant's life. Some studies confirmed a significant association between the low level of mothers' education and breastfeeding cessation before the 6 months of an infant's life (11–14). Mother's attitude toward the breastfeeding practice can be shaped by area of living, type of settlement, aesthetic reasons that are becoming more and more frequent in the population of future mothers (15).

Ethnicity can influence the practice of exclusive breastfeeding, especially belonging to the Roma population. The Roma population is the second-largest ethnic minority in Serbia, and according to official data, there are almost 150,000 Roma, which is 2.05% of the total population in the Republic of Serbia (16). The Roma population is among the most vulnerable population group in Europe, especially in the Republic of Serbia, quite often exposed to discrimination, social marginalization and poverty, but also with less access to health services, especially mother and child to preventive health services and practice (17–20).

According to the last available data, the infant mortality rate in the Republic of Serbia is 5 per 1,000 live births in the non-Roma population (inter agency groups) while in Roma population it accounts for 12.8 per 1,000 live births (21). When it comes to the under-five mortality rate, in non-Roma population it is 6 in the non-Roma population (22) and 14.4 in Roma population (21). Although under-five mortality in Serbia has reduced in recent years for both populations is below the Sustainable Development Goal target of 25 deaths per 1,000 live births (23), it is still important to highlight the importance of breastfeeding for the improvement of children outcomes (1, 2).

Data from Multiple Indicators Cluster Surveys (MICS) that have been periodically conducted in Serbia (21, 24) showed that the prevalence of child undernutrition (under the age of 5) in recent year was higher than earlier in Roma children while there was a slight decrease in undernutrition rate in non-Roma children (13 vs. 12% for non-Roma children and 31 vs. 32.8% for Roma children) (21, 24). Still, it is important to make an effort to prevent childhood malnutrition and the prevention of breastfeeding as one of the crucial activities in this field.

Numerous studies around the world examined the factors influencing the breastfeeding practice, but studies of this type are not frequent among vulnerable populations, such as Roma community. Therefore, our analysis aimed to assess the prevalence of exclusive breastfeeding practice and identify the potential factors associated with the practice of exclusive breastfeeding of infants up to 6 months on representative samples of the Roma and non-Roma population.

## Materials and Methods

### Population and Sampling

The study is secondary data analyses of the last available Multiple Indicators Cluster Survey (MICS) conducted in 2014. The survey is the fifth round (MICS5) of these studies performed by the Statistical Office of the Republic of Serbia with the technical support of UNICEF since 1995 (21). Since this is a secondary analysis, the process on data abstraction technique followed the research protocol the team developed and has gone through the development of the research questions, the identification of the dataset, and thorough evaluation the dataset to ensure the appropriateness for the Research Topic.

MICS 5 included the two national representative samples of the general non-Roma population and the Roma population living in Roma settlements in Serbia. Both samples were planned to provide the assessment of a large number of the indicators of the mothers and children characteristics at the national level, for the urban and rural area, and four regions: Belgrade (the capital city), Vojvodina, Sumadija and Western Serbia, and the region of Southern and Eastern Serbia. Urban and rural settlements within each region were defined as the main sample strata. Within each stratum, a certain number of enumeration circles were selected systematically with a probability of a proportional sample size. Later, all households were divided into households with and without children under the age of five and a separate systematic sample of households was selected for each group.

According to this recruitment method, a total of 1976 Roma households were identified, and 1,743 were found to be populated and approached by the interview (household response rate was 97%). In the interviewed households, 1,556 children under the age of 5 years were identified. Out of that number, for 1,515 children, the questionnaire was filled by the mother or caretakers, giving a response rate of 97% within the interviewed households.

According to this recruitment method, a total of 7,351 non-Roma households were identified, and 6,191 were found to be populated and approached by the interview (household response rate was 89%). In the interviewed households, 2,773 children under the age of 5 years were identified. Out of that number, for 2,720 children, the questionnaire was filled by the mother or caretakers, giving a response rate of 98.1% within the interviewed households.

The sample for research Serbia MICS 2014 was weighted to present the results at the national level. This analysis includes 146 Roma and 321 non-Roma infants who present all children under the age of 6 months whose mothers filled in the questionnaires in given sample.

### Survey Instruments

During the research, three types of questionnaires were used:

(1) household questionnaire for collecting data on demographic and socio-economic characteristics of the household.
(2) a questionnaire for women filled in each household with all women aged 15–49 for obtaining data on characteristics of the mothers; and
(3) a questionnaire for children under the age of five filled by mothers or primary caretakers for obtaining the information about the infant.

All questionnaires are based on the standard versions of MICS questionnaires. The questionnaires were translated into Serbian and pre-tested. Based on the results of the pre-test, modifications were made to the wording and translation of the final version of questionnaires. The data from the non-Roma sample were collected by 15 teams; each was comprised of two female interviewers, one female editor, one male interviewer/measurer/driver and a supervisor. On the other hand, the data from the Roma settlements sample were collected by three teams. Each team was comprised of two female Roma interviewers, one female editor, one male interviewer/measurer/driver and a supervisor.

### Variables

The evidence from the literature informed the selection of all variables, as well as the researchers' opinions on their significance. The variables used in the research were related to family, child and mother characteristics.

The family characteristics included the following variables: type of settlement (urban/rural), region (Belgrade, Vojvodina, Sumadija and Western Serbia and southern and eastern Serbia), marital status (life with partner/life without a partner), the age of the mother and the age of a partner. Based on the values of the wealth index, respondents were classified into the five groups—quintiles: the first one (the poorest), the second (poor), the third (middle), the fourth (rich), and the fifth (the richest). This wealth index was calculated by the principal components analysis (PCA) using the variables related to the possession of examinees' assets, characteristics of the house, water and disposal of waste materials, as well as other characteristics associated with the well-being of the household (25).

The mother's characteristics included: the level of education (without education/elementary school/secondary school/higher and high school), mother's age, parity (primipara/multipara), previous abortion (yes/no), the desire for pregnancy (yes/no), attending a childbirth preparation program at a health institution (yes/no), and establishing a menstrual cycle after delivery (yes/no).

The infant's characteristics included: infant age in months, sex (male/female), birth weight (<2,500 g/2,500 g and more), type of delivery (natural/cesarean section), staying in the same room with the mother (yes/no), whether the baby at the maternity ward drank something other than breast milk in the first 3 days after birth (yes/no) and the breastfeeding initiation (in the first hour after delivery/after the first hour).

The practice of exclusively breastfeeding refers to infants younger than 6 months of life who, in addition to breast milk, could only get vitamins, extra minerals and medicines (2). The exclusively breastfed baby was identified based on taking the different types of fluids and foods during the previous day, as well as on the fact that the mother is still breastfeeding.

In order to avoid recall bias, the research questions were carefully defined and of high quality. The information on breastfeeding that mothers provided were related to the previous day so they only had to recall the short-term memory. Also, the interviews were well trained before their fieldwork which is inherent in MICS methodology.

### Statistical Analysis

Chi-square, Student *T*-test and logistic regression analyses served for data analysis. A comparison between two groups (those who exclusive breastfed and those who did not) was done concerning the family, mother's and infant's characteristics. The associations between dependent (the practice of exclusive breastfeeding) and independent variables were examined by using the univariate (ULRA) and multivariate logistic regression analysis (MLRA). All independent variables, whose *p*-values in the univariate logistic regression were <0.05, were included in the multivariate logistic regression models. The results (95% confidence interval) in both univariate and multivariate logistic regression were considered significant if the *p*-value was < 0.05 in the final model. The analyses were done by using the statistical software package SPSS 20 (IBM Corp. Released 2011. IBM SPSS Statistics for Windows, Version 20.0. Armonk, NY: IBM Corp.).

## Results

The study included mothers of 467 infants under 6 months of age. The percentage of exclusively breastfed babies is 13.3%, with no significant difference for ethnicity (Roma population 13%, non-Roma population 13.4%, *p* = 0.910).

### Demographic and Socio-Economic Characteristics of the Populations

Table 1 is presenting the demographic and socio-economic characteristics of the family. In the Roma population, every fifth infant up to 6 months living in Belgrade is exclusively breastfed, every seventh in Vojvodina, and every tenth in southern and eastern Serbia. In the non-Roma population, every third infant in Belgrade is exclusively breastfed, while one in 100 babies in southern and eastern Serbia is exclusively breastfed. In both populations, there were significantly more exclusively breastfed babies in urban than in rural areas (Roma: 15.9 vs. 5.1%, *p* = 0.102; non-Roma: 19.2 vs. 1.8%, *p* < 0.001). In both populations, the practice of exclusive breastfeeding is most prevalent among wealthier families (Table 1).

**Table 1:** Family characteristics and practice of exclusive breastfeeding under the age of 6 months.

	**Roma population**				**Non-Roma population**			
**Family characteristics**	**Exclusive breastfeeding**		**Total *n* (%) 146 (100.00)**	*p*-value^*^	**Exclusive breastfeeding**		**Total *n* (%)** **321 (100.00)**	*p*-value^*^
	**Yes *n* (%)** **19 (13.0)**	**No *n* (%)** **127 (87.0)**			**Yes *n* (%)** **43 (13.4)**	**No *n* (%)** **278 (86.6)**		
Region				0.510				<0.001
Beograd	7 (20.0)	28 (80.0)	35 (24.0)		23 (34.3)	44 (65.7)	67 (20.9)	
Vojvodina	3 (13.6)	19 (86.4)	22 (15.1)		14 (11.38)	109 (88.6)	123 (38.4)	
Šumadija i zapadna Srbija	2 (8.0)	23 (92.0)	25 (17.1)		5 (11.1)	40 (88.9)	45 (14.1)	
JuŽna i istočna Srbija	7 (10.9)	57 (89.1)	64 (43.8)		1 (1.2)	84 (98.8)	85 (26.6)	
Area				0.102				<0.001
Rural	2 (5.1)	37 (94.9)	39 (26.7)		2 (1.8)	106 (98.2)	108 (33.6)	
Urban	17 (15.9)	90 (84.1)	107 (73.3)		41 (19.2)	172 (80.8)	213 (66.4)	
Marital status				0.076				1.000
Living with a partner	19 (15.2)	106 (84.8)	125 (85.6)		43 (15.7)	274 (86.4)	317 (98.8)	
Living without a partner	0 (0)	21 (100.0)	21 (14.4)		0 (0)	4 (100.0)	4 (1.2)	
Wealth index				0.003				<0.001
Poorest	6 (16.7)	30 (83.3)	36 (24.7)		0 (0)	26 (100.0)	26 (8.1)	
Poor	2 (7.4)	25 (92.6)	27 (18.5)		1 (3.0)	32 (97.0)	33 (10.3)	
Middle	0 (0)	43 (100.0)	43 (29.5)		7 (9.0)	71 (91.0)	78 (24.3)	
Rich	5 (33.3)	10 (66.7)	15 (10.3)		6 (6.5)	87 (93.5)	93 (29.0)	
Richest	6 (24.0)	19 (76.0)	25 (17.1)		29 (31.9)	62 (68.1)	91 (28.3)	

* *According to chi-square test*.

The average age of mothers who exclusively breastfed their babies is 22 years (SD ± 4.91) in the Roma population and 30 years (SD ± 3.87) in the non-Roma population (data not shown in the table). The average age of fathers of exclusively breastfed infants is 25 years (SD ± 6.28) in the Roma population and 36 years (SD ± 10.09) in the non-Roma population. While in Roma population no statistically significant difference has been observed between the practice of exclusive breastfeeding and father's age (*p* = 0.127), a significant difference between the practice of exclusive breastfeeding and father's age has been found (*p* = 0.002) (data not shown in the table).

### Infant Characteristics and Exclusive Breastfeeding Practices

The characteristics of infants are shown in Table 2. The distribution of infants by sex was relatively uniform in both populations (Roma population: boys 58.9%, girls 41.1%; non-Roma population: boys 51.1%, girls 48.9 %). The average age of exclusively breastfed infants in months has identical values in both populations and is 2 months with a standard deviation of 1.55 (not shown in the table). Boys were more often exclusively breastfed than girls (Roma population: 14.0 vs. 11.7%, *p* = 0.805; non-Roma population: 22.3 vs. 4.9%, *p* < 0.001). In both populations, all infants exclusively breastfed were over 2,500 g at birth.

**Table 2:** Infant's characteristics and practice of exclusive breastfeeding under the age of 6 months.

	**Roma population**				**Non-Roma population**			
**Infants characteristics**	**Exclusive breastfeeding**		**Total *n* (%)** **146 (100.00)**	*p*-value^*^	**Exclusive breastfeeding**		**Total *n* (%)** **321 (100.00)**	*p*-value^*^
	**Yes *n* (%)** **19 (13.0)**	**No *n* (%) 127 (87.0)**			**Yes *n* (%)** **43 (13.4)**	**No *n* (%)** **278 (86.6)**		
Sex				0.805				< 0.001
Girls	7 (11.7)	53 (88.3)	60 (41.1)		8 (4.9)	156 (95.1)	164 (48.9)	
Boys	12 (14.0)	74 (86.0)	86 (58.9)		35 (22.3)	122 (77.7)	157 (51.1)	
Birth weight				0.013				0.054
<2,500 g	0 (0)	33 (100.0)	33 (22.9)		0 (0)	22 (100.0)	22 (7.0)	
2,500 g and more	18 (16.2)	93 (83.3)	111 (77.1)		43 (14.8)	248 (85.2)	291 (93.0)	
Type of delivery				0.922				0.360
Natural	16 (12.6)	111 (87.4)	127 (88.2)		34 (15.0)	192 (85.0)	226 (72.2)	
Cesarian section	2 (11.8)	15 (88.2)	17 (11.8)		9 (10.3)	78 (89.7)	87 (27.8)	
Breastfeeding initiation				0.196				1.000
Within the first hour	16 (16.5)	81 (83.5)	97 (68.8)		141 (86.5)	22 (13.5)	163 (50.6)	
Later than the first hour	3 (6.8)	41 (93.2)	44 (31.2)		136 (86.1)	22 (13.9)	158 (49.4)	
A baby drank something other than mother's milk in the first 3 days after birth?				1.000				0.037
Yes	7 (13.5)	45 (86.5)	52 (36.9)		24 (11.6)	183 (88.4)	207 (70.4)	
No	12 (13.5)	77 (86.5)	89 (63.1)		19 (21.8)	68 (78.2)	87 (29.6)	
A baby stayed in the same room with a mother during the stay in the hospital?				1.000				0.012
Yes	14 (12.7)	96 (87.3)	110 (75.3)		20 (10.0)	180 (90.0)	200 (63.5)	
No	5 (13.9)	31 (86.1)	36 (24.7)		24 (20.9)	91 (79.1)	115 (36.5)	

* *According to chi-square test*.

In the Roma population, there was a higher prevalence of the practice of exclusive breastfeeding among mothers who made the first attempt to breastfeed within the first hour after delivery than those who attempted breastfeeding later (16.5 vs. 6.8%). No such difference was observed in the non-Roma population (86.5 vs. 86.1%).

While there was no statistically significant difference between the breastfeeding practice and keeping mother and baby in the same room after the delivery in the Roma population, a statistically significant difference was observed in the non-Roma population. In the non-Roma population, there were less exclusively breastfed babies among those who stayed in the same room compared to those who did not stay in the same room (20.9 vs. 10.0%, *p* = 0.012) (Table 2).

### Mother's Characteristics and the Practice of Exclusive Breastfeeding

The mother's characteristics and the practice of exclusive breastfeeding are shown in Table 3. In the Roma population, there was no significant difference between the practice of exclusive breastfeeding and the level of maternal education. However, in the non-Roma population, a significantly higher percentage of mothers who exclusively breastfed their babies had higher or high school completed.

**Table 3:** Mother's characteristics and practice of exclusive breastfeeding under the age of 6 months.

	**Roma population**				**Non-Roma population**			
**Mother's characteristics**	**Exclusive breastfeeding**		**Total *n* (%)** **146 (100.00)**	*p*-value^*^	**Exclusive breastfeeding**		**Total *n* (%)** **321 (100.00)**	*p*-value^*^
	**Yes *n* (%)** **19 (13.0)**	**No *n* (%)** **127 (87.0)**			**Yes *n* (%)** **43 (13.4)**	**No *n* (%) 278 (86.6)**		
Level of education				0.968				0.003
No education and primary	3 (11.5)	23 (88.5)	26 (17.8)		0 (0)	27 (100.0)	27 (8.4)	
Secondary	15 (13.4)	97 (86.6)	112 (76.7)		18 (11.1)	143 (88.8)	161 (50.2)	
Higher/high	1 (12.5)	7 (87.5)	8 (5.5)		25 (18.8)	108 (81.2)	133 (41.4)	
Parity				0.041				<0.001
Primipara	10 (21.3)	37 (78.7)	47 (32.2)		6 (4.61)	130 (95.6)	136 (42.2)	
Multipara	9 (9.1)	90 (90.9)	99 (67.8)		37 (27.2)	99 (72.8)	136 (57.8)	
Pevious miscarriages				0.007				0.020
No	1 (1.9)	51 (98.1)	52 (35.6)		25 (10.0)	224 (90.0)	249 (77.6)	
Yes	18 (19.1)	76 (80.9)	94 (64.4)		18 (25.0)	54 (75.0)	72 (22.4)	
Desire for the last birth				0.428				0.110
No	1 (4.8)	20 (95.2)	21 (14.5)		1(3.1)	31 (96.9)	32 (10.2)	
Yes	17 (13.7)	107 (86.3)	124 (85.5)		43 (15.2)	240 (84.8)	283 (89.8)	
Attending a childbirth preparation programme				-				<0.001
No	19 (13.0)	127 (87.0)	146 (100.0)		28 (10.1)	248 (89.9)	276 (87.9)	
Yes	0 (0)	0 (0)	0 (0)		15 (39.5)	23 (60.5)	38 (12.1)	
Menstrual period has returned since the child birth				0.041				<0.001
Yes	8 (8.4)	87 (91.6)	95 (65.1)		4 (3.5)	110 (96.5)	114 (36.2)	
No	11 (22.0)	39 (78.0)	50 (34.2)		40 (19.9)	161 (80.1)	201 (63.8)	

* *According to chi-square test*.

While mothers primipara in Roma population more often exclusively breastfed their babies than multipara (21.3 vs. 9.1%, *p* = 0.041), the opposite pattern has been noted in non-Roma mothers (4.61 vs. 27.2%, *p* < 0.001). There were none of the Roma women that attended a childbirth preparation programme (pregnancy and parenting education in primary health care institutions) at a healthcare facility. On the other hand, non-Roma mothers who attended a childbirth preparation program more frequently exclusively breastfed their child than those who did not attend (39.5 vs. 10.1%, *p* < 0.001).

In both populations, there were significantly more exclusively breastfed infants in the group of mothers whose menstrual period did not return since the childbirth compared to those whose menstrual period returned (Table 3).

### The Association of Different Factors and the Practice of Exclusive Breastfeeding

Tables 4–6 show the association between demographic and socio-economic characteristics of the family, infant's characteristics and mother's characteristics with the practice of exclusive breastfeeding during the first 6 months of an infant's life. All variables that proved to be significant in the univariate logistic regression analyses were included in the final model of multivariate logistic regression (Table 7).

**Table 4:** The associations between demographic and socio-economic characteristics of the family and the practice of exclusive breastfeeding—Univariate logistic regression (ULRA).

	**Roma population**		**Non-Roma population**	
**Family characteristics**	**Univariate logistic**		**Univariate logistic**	
	**regression analyses**		**regression analyses**	
	***p*-value**	**OR (95% CI)**	***p*-value**	**OR (95% CI)**
**Region**				
Beograd		1.00		1.00
Vojvodina	0.503	0.61 (0.14–2.60)	<0.001	0.25 (0.12–0.52)
Šumadija i zapadna Srbija	0.162	0.29 (0.05–1.65)	0.008	0.24 (0.08–0.69)
JuŽna i istočna Srbija	0.192	0.47 (0.15–1.46)	0.001	0.02 (0.01–0.17)
**Area**				
Rural		1.00		1.00
Urban	0.134	3.01 (0.71–12.73)	0.001	10.58 (2.80–40.06)
**Marital status**				
Living with a partner		1.00		1.00
Living without a partner	0.998	-	0.999	-
**Wealth index**				
Poorest		1.00		1.00
Poor	0.338	0.44 (0.08–2.37)	0.998	0.00
Middle	0.997	-	0.009	0.07 (0.01–0.52)
Rich	0.123	2.97 (0.74–11.88)	0.001	0.22 (0.09–0.52)
Richest	0.449	1.64 (0.45–5.97)	<0.001	0.14 (0.56–0.37)
Father's age	0.149	0.94 (0.87–1.02)	0.011	1.06 (1.01–1.11)

**Table 5:** The associations between infants' characteristics and the practice of exclusive breastfeeding—Univariate logistic regression.

	**Roma population**		**Non-Roma population**	
	***P*-value**	**OR (95% CI)**	***P*-value**	**OR (95% CI)**
**Sex**				
Girls		1.00		1.00
Boys	0.684	1.23 (0.45–3.34)	<0.001	5.41 (2.44–12.00)
Age (months)	0.104	0.74 (0.52–1.06)	0.044	0.80 (0.64–0.99)
**Type of delivery**				
Natural		1.00		1.00
Cesarian section	0.858	1.16 (0.24–5.66)	0.244	1.60 (0.73–3.51)
**Breastfeeding initiation**				
Within the first hour		1.00		1.00
Later than the first hour	0.093	0.31 (0.08–1.21)	0.950	1.02 (0.54–1.94)
**A baby drank something other than mother's milk in the first 3 days after birth?**				
Yes		1.00		1.00
No	0.947	0.97 (0.36–2.63)	0.031	2.08 (1.07–4.03)
**A baby stayed in the same room with a mother during the stay in the hospital?**				
Yes		1.00		1.00
No	0.834	1.12 (0.37–3.38)	0.009	2.37 (1.24–4.55)

**Table 6:** The associations between mothers' characteristics and the practice of exclusive breastfeeding—Univariate logistic regression.

	**Roma population**		**Non-Roma population**	
	***P*-value**	**OR (95% CI)**	***P*-value**	**OR (95% CI)**
**Level of education**				
No education		1.00		
Primary^a^	0.783	1.20 (0.32–4.52)		1.00
Secondary^b^	0.928	1.12 (0.10–13.06)	0.998	1.20 (0.32–4.52)
Higher/high			0.998	1.12 (0.10–13.06)
Age	0.170	0.94 (0.85–1.03)	0.085	0.94 (0.85–1.03)
**Parity**				
Primipara		1.00		1.00
Multipara	0.066	0.40 (0.15–1.06)	<0.001	6.17 (2.48–15.33)
**Pevious abortions**				
No		1.00		1.00
Yes	0.431	0.20 (0.01–10.84)	0.001	3.09 (1.58–6.06)
**Desire for the last birth**				
No		1.00		1.00
Yes	0.363	2.24 (0.40–12.68)	0.093	9.84 (0.68–142.06)
**Attending a childbirth preparation programme**				
No	NA	NA		1.00
Yes			<0.001	6.05 (2.84–12.90)
**Menstrual period has returned since the child birth**				
Yes		1.00		1.00
No	0.016	3.40 (1.26–9.18)	<0.001	7.53 (2.49–22.80)

a *only for non-Roma population: no education and primary education are compressed into one category*.

b *only for Roma population: secondary and higher/high education are compressed into one category*.

**Table 7:** Potential predictors of exclusive breastfeeding practice during the first 6 months adjusted for mothers' and infants' age—final model by multivariate logistic regression (MLRA).

	**Roma population**		**Non-Roma population**	
	***P*-value**	**OR (95% CI)**	***P*-value**	**OR (95% CI)**
**Region**				
Beograd				1.00
Vojvodina			**0.040**	**0.16 (0.03–0.92)**
Šumadija i zapadna Srbija			0.430	0.49 (0.08–2.86)
JuŽna i istočna Srbija			**0.007**	**0.02 (0.01–0.35)**
**Area**				
Rural				1.00
Urban			**0.006**	**10.35 (1.94–55.28)**
Father's age			**0.019**	**1.07 (1.01–1.13)**
**Gender**				
Girls				1.00
Boys			0.840	1.14 (0.32–4.00)
**A baby drank something other than mother's milk in the first 3 days after birth?**				
Yes				1.00
No			** <0.002**	**10.14 (2.28–45.18)**
A baby stayed in the same room with a mother during the stay in the hospital?				
Yes				1.00
No			**0.005**	**7.19 (1.80–28.68)**
**Parity**				
Primipara		1.00		1.00
Multipara	0.114	0.42 (0.14–1.23)	**0.038**	**4.78 (1.09–20.93)**
**Breastfeeding initiation**				
Within the first hour		1.00		
Later than the first hour	0.143	0.34 (0.08–1.44)		
**Attending a childbirth preparation programme**				
No				1.00
Yes			**0.023**	**8.47 (1.34–53.67)**
**Menstrual period has returned since the child birth**				
Yes		1.00		1.00
No	**0.014**	**3.90 (1.32–11.56)**	0.122	3.36 (0.72–15.57)

In the Roma population, ULRA does not show an association of exclusive breastfeeding practice neither with any demographic and social-economic characteristics of the household nor infant's characteristics. However, in the non-Roma population, there is a significant association between all examined demographic and social-economic variables (except marital status) and exclusive breastfeeding practices (Table 4). However, in the non-Roma population, there is a significant association between practice of exclusive breastfeeding and the sex of infants, the age of the baby in months, the fact whether the baby in the hospital drank anything other than breast milk in the first 3 days after birth, and the stay of the mother and baby in the same room after the delivery. In the non-Roma population, boys are more than 5 times prone to be exclusively breastfed than girls (OR 5.41, CI 95% 2.44–12.00). Infants who did not stay in the same room with mothers were 2.5 times more likely to be exclusively breastfed (OR 2.37, CI 95% 1.24–4.55) compared to those who stayed in separate rooms. Although without statistical significance, this pattern is also present in the Roma population (Table 5). While in Roma population the associations between the practice of exclusive breastfeeding and mothers' characteristics were not observed, in non-Roma population multiparity, the existence of previous miscarriage, attendance of childbirth preparation program and menstrual cycle return significantly increased the chance of infant to be exclusively breastfed (Table 6).

After performing the MLRA, in the Roma population, only the menstrual cycle return proved to increase the likelihood of infants to be exclusively breastfed (OR 3.90, CI 95% 1.32–11.56). However, in non-Roma population region, area of living, father's age, the fact whether the baby in the hospital drank anything other than breast milk in the first 3 days after birth, the stay of the mother and baby in the same room after the delivery, parity and attendance of childbirth preparation program have showed significant associations with the practice of exclusive breastfeeding (Table 7).

## Discussion

This analysis is the first study in the Republic of Serbia conducted on a national sample of non-Roma and Roma populations that examined potential predictors of exclusive breastfeeding. A tiny percentage (13.3%) of exclusively breastfed infants under 6 months, with almost no difference between the populations, indicates to the neglected importance of breast milk as a food during the first months of life. A series of social changes during the 20th and 21st centuries such as greater mobility of families, the popularization of infant milk formulas, a passive attitude of the community and health care professionals toward natural nutrition, led to the rapid takeover of adapted milk formula over breastfeeding (3, 26).

Compared to the neighboring countries, the lowest percentage of exclusively breastfed infants has been observed in the Republic of Serbia. Only in the Republic of Serbia, the percentage of exclusively breastfed infants does not differ between the Roma and non-Roma population, while in other neighboring countries, Roma population has a higher frequency of exclusive breastfeeding practice than non-Roma population (Bosnia and Herzegovina: 22.3% vs. 18.3 %; Northern Macedonia 32.1% and 23.0%) (27, 28). These findings of significantly low rates of exclusive breastfeeding in the Republic of Serbia serve as an alarm that prevention work needs an urgent intervention to educate both healthcare professionals and mothers who are at risk of not breastfeeding or stopping breastfeeding before the recommended times, regardless the ethnicity.

According to the results of our study, infants from the capital, Belgrade, had the best chance to be exclusively breastfed for the first 6 months of age in both populations. Data from the Statistical Office of the Republic of Serbia on income in money and kind and individual consumption of households show that the highest revenues are in Belgrade, while the lowest are in Southern and Eastern Serbia. The following pattern has appeared: the better income indicators were observed, the higher the percentage of exclusively breastfed infants was. Hence, places where living and working conditions are better and the incomes are higher create a supportive environment for women to breastfeed (29).

Furthermore, the highest percentage of the exclusively breastfed infant in the capital partly could be a result of the existence and the activities of the Hallo Baby phone counseling center launched in Belgrade in 2001 within the Health Promotion Center, City Institute of Public Health. This counseling center contributes to the promotion of the mothers' and child health by counseling and encouraging decision-making related to health, proper nutrition, care, growth and development of children, based on adequate and timely information and advice under professional evidence. This service is available 24/7 and provides support to all parents from the capital who mostly calls when the child is sick, but also when the breastfeeding is about (30). Moreover, the biggest efforts to organize childbirth preparation programs within health institutions have been made in Belgrade during the last several years, so it is not surprising that mothers from Belgrade are strengthened by knowledge in this field.

The practice of exclusive breastfeeding in urban areas was more common than in rural areas. Rural areas are characterized by low population rates, low birth rates, so significant investments are needed to support mothers to breastfeed. Equal access and accessibility issues are of crucial importance when it comes to the broader picture of breastfeeding support in any region, especially in rural areas. Study on factors associated with the beginning and duration of breastfeeding in rural areas in the United States has shown that economic factors in rural areas, such as the woman need to return to work and limited resources to support the continuation of breastfeeding, represent significant barriers to the practice of exclusive breastfeeding (31–33). The results of our study are in accordance with the studies done worldwide (31–33) and there is no doubt that mothers living in urban areas benefit from the density and variety of support services and breastfeeding expertise (34).

Women living in rural areas are often unemployed due to lack of qualifications and low level of education, they do not have social interaction with other women because they stay at home while partner makes a living, which altogether leads to a lack of knowledge about breastfeeding and its importance especially during the first months of infant's life. Women's rights are less respected in such environments where women stay home and care for children, and men earn money (35). Therefore, special attention should be paid to raising the awareness of parents who are geographically or culturally isolated in rural areas regarding breastfeeding.

While the age of the father was not associated with the practice of exclusive breastfeeding in the Roma population, in the non-Roma population, the likelihood that the mother will exclusively breastfeed the infant for the first 6 months of life increases with the age of the partner. The assumption is that older men are more mature, aware of the benefits for their child and as such an essential support for women during the first months of the infant's life. Wambach showed that women who exclusively breastfeed, succeeded because their partners supported the decision, believing that breastfeeding was best for the baby (36). Partner contributes to the practical and emotional support for breastfeeding and several studies have found that greater father involvement during the prenatal period increases the likelihood of breastfeeding initiation and duration (37, 38). The support of fathers maybe even more influential especially in a rural area where women may be geographically isolated from their mothers or other family members (37, 38).

Although studies conducted worldwide have shown that the Baby-Friendly Program has significantly increased the rate of exclusive breastfeeding in the world (39, 40), the practice of infant staying in the same room with the mother in the maternity ward showed, in contrast, quite opposite results. In the Roma population, mothers who did not stay in the same room with their baby had a seven-fold higher chance of breastfeeding exclusively during the first 6 months, whereas in the Roma population staying in the same room did not affect the practice of breastfeeding. The assumption is that a larger sample of Roma population would give a similar result since the values of logistic regression were going in the same direction. One of the explanations for these findings is found in the evaluation of the Baby-Friendly Program in the Republic of Serbia, showing that the Baby-Friendly Program was not implemented properly (41). On the other hand, mothers were frustrated by the fact that they were alone with their newborn for a considerable part of the time, did not cope with breastfeeding, especially the primipara and there was insufficient help regarding breastfeeding. While babies were crying, mothers did not know how to behave; they waited for a long time for someone to show up and help them. During this time, those mothers not included in Baby-Friendly Program are at the regular regime of sleep, rest, they do not hear crying, babies are fed and those mothers later leave the hospital more relaxed and rested (41). Thus, much greater support is needed for mothers, especially encouraging them to start breastfeeding in the first hour after birth and to stay together with the baby 24 h a day (7, 8). In 2012, the Republican Expert Commission (REC) on Health Protection of Women, Children and Youth adopted “National standards of health care tailored to the needs of mothers and children” which provides the same standard of service and the right of infants to be fed by breast milk in all health care institutions. However, in 2014 the REC Perinatal Health Task Force surveyed maternity wards and found that the implementation of the National Standards was incomplete in almost all health care facilities. One example is the application of adapted milk formula in maternity wards without medical records (42).

Studies worldwide have shown that attending antenatal breastfeeding educations is one of the crucial steps for breastfeeding success (43–46). Pregnant women with knowledge about breastfeeding are more likely to plan breastfeeding, which is key to following breastfeeding recommendations later (47–49). Regarding this, attending a childbirth preparation program (unique educational program on pregnancy, nutrition, physical activity, childbirth, breastfeeding, mother care and newborn child within health care institutions) can significantly improve maternal knowledge on pregnancy health, breastfeeding, newborn care and parenting skills. In our study, non-Roma mothers attending the childbirth preparation program were 18 times more likely to exclusively breastfeed their baby. However, the problem here is an extremely low number of mothers attending the birth preparation program. One of the reasons for the low attendance rate is that this form of counseling work is available in 40 out of 160 existing health centers established by the Regulation on the Network of Health Care Facilities Network Plan (50). Also, women from the Roma population are significantly less likely to undergo regular checkups during pregnancy. Very often their first visit is for childbirth so their absence can be associated with the lack of knowledge about the existence of these types of support (51).

Although in Roma population no correlation was observed between the number of children and the practice of exclusive breastfeeding, in the non-Roma population, the likelihood for mothers to exclusively breastfeed was higher with each subsequent child. A positive association between the practice of exclusive breastfeeding and the number of children has been confirmed in studies by Radwan, as well as by Yilmaz where mothers of two or more children were found to be more likely to exclusively breastfeed compared to those with only one child (52, 53). Failure to start breastfeeding with the first child is associated with failure to start and continue breastfeeding with the second and/or subsequent child (54, 55). Jessri confirmed that mothers multipara were more than twice as likely to exclusively breastfeed their babies for the first 6 months than primipara (56). Women who gave birth at least twice had more knowledge, confidence and experience, and were more likely to exclusively breastfeed their infant for 6 months (57). In this regard, the importance of counseling and developing breastfeeding skills for first-borns should be emphasized in order to overcome barriers and to establish, promote and support breastfeeding.

What characterizes women in both populations is the association between the practice of exclusive breastfeeding and the return of the menstrual cycle. If a menstrual cycle has not returned after childbirth, that woman is more likely to breastfeed longer. A study in Nigeria has shown that breastfeeding duration is the most significant predictor of the duration of lactational amenorrhea (58). Although lactational amenorrhea is used as a method of contraception, it is important to emphasize that it does not provide protection again pregnancy by itself. According to The Bellagio Consensus, the necessary conditions under which breastfeeding can be safely and effectively used as a contraceptive method are lactational amenorrhea, exclusive breastfeeding, and a period shorter than 6 months after birth (59).

### Limitations and Advantages of the Study

A cross-sectional study design makes impossible the examination of the causal relationships between predictors and outcomes of interest (exclusive breastfeeding practices). Also, the study design is limited by potential bias because exclusive breastfeeding data depends on mothers' memories.

The important advantage of this study is that examines factors related to the practice of exclusive breastfeeding on a representative sample of Roma as a vulnerable community and average non-Roma population. Also, the study covered several types of indicators (indicators relating to family, mother and infants) so we were able to explore which of the given categories has the greatest impact on exclusive breastfeeding practice. The results could be used for the breastfeeding education programmes development. Further studies should focus on identifying knowledge on breastfeeding, how it influences the breastfeeding practice and how to focus additional interventions on the development, introduction, and evaluation of the breastfeeding support programs. Understanding the predictors of exclusive breastfeeding can help create programs that promote exclusive breastfeeding practices for the first 6 months and can lead to more infants being exclusively breastfed in accordance with WHO guidelines.

## Conclusion

The prevalence of exclusive breastfeeding of infants younger than 6 months was 13.3%, with no difference between Roma and non-Roma population, which is far below the WHO recommendations. While in Roma population the choice of a woman to exclusively breastfeed is associated with the duration of lactational amenorrhea, in general population the decision of a woman to exclusively breastfeed is strongly associated with the region where she lives, the type of settlement, the age of the partner, the fact whether the baby drank anything other than breast milk days in the maternity ward, staying in the same room with the mother at the hospital, parity, and attending the birth preparation program during pregnancy.

Preventive work should focus on improving the knowledge (education) of healthcare professionals and mothers who are at risk of stopping breastfeeding before the recommended time. Also, it is necessary to urgently start with the implementation of all 10 steps BFHI in maternity wards, mobilizing resources, and providing support to mothers who have breastfeeding problems regardless of the region and type of settlement.

## Data Availability Statement

The datasets generated for this study are available on request to the corresponding author.

## Ethics Statement

The studies involving human participants were reviewed and approved by UNICEF (Multiple Claster Indicator Study). Written informed consent to participate in this study was provided by the participants' legal guardian/next of kin.

## Author Contributions

ZS contributed to concept and design, acquisition of data, analysis and interpretation of data, drafting the article, and final approval of the manuscript. BM contributed to concept and design, analysis of data, revising the article draft critically for important intellectual content, and final approval of the manuscript. BD contributed to concept and design, revising the article draft critically for important intellectual content, and final approval of the manuscript. VB-M contributed to concept and design, revising the article draft critically for important intellectual content, and final approval of the manuscript. All authors agree to take responsibilities for all aspects of the work in ensuring that questions related to the accuracy or integrity of any part of the work are appropriately investigated and resolved, accept responsibility for the paper as published, and took part in this research from the very early beginning until the final approval of the manuscript.

## Conflict of Interest

The authors declare that the research was conducted in the absence of any commercial or financial relationships that could be construed as a potential conflict of interest.
