# Corrigendum: Surprising Differences in the Practice of Exclusive Breastfeeding in Non-Roma and Roma Population in Serbia

**DOI:** 10.3389/fpubh.2021.652891

**Published:** 2021-02-10

**Authors:** Zeljka Stamenkovic, Bojana Matejic, Bosiljka Djikanovic, Vesna Bjegovic-Mikanovic

**Affiliations:** Institute of Social Medicine, Medical Faculty University of Belgrade, Belgrade, Serbia

**Keywords:** breastfeeding, Roma, non-Roma, baby friendly, childbirth preparation program

There is an error in the Acknowledgment statement. The correct number for project number is 175042.

The authors apologize for this error and state that this does not change the scientific conclusions of the article in any way. The original article has been updated.

